# The role of comorbidities in a rare case of sequential atraumatic lateral malleolus fractures

**DOI:** 10.1093/jscr/rjaf437

**Published:** 2025-06-23

**Authors:** Danil V Chernov, Nicholas Frappa, Matthew G Alben, Joshua Slowinski, Ryan Riley, Jennifer Gurske-dePerio

**Affiliations:** Jacobs School of Medicine and Biomedical Sciences, 955 Main Street, Buffalo, NY 14203, United States; Jacobs School of Medicine and Biomedical Sciences, 955 Main Street, Buffalo, NY 14203, United States; University at Buffalo, Department of Orthopaedics and Sports Medicine, 462 Grider Street, Buffalo, NY 14215, United States; University at Buffalo, Department of Orthopaedics and Sports Medicine, 462 Grider Street, Buffalo, NY 14215, United States; University at Buffalo, Department of Orthopaedics and Sports Medicine, 462 Grider Street, Buffalo, NY 14215, United States; University at Buffalo, Department of Orthopaedics and Sports Medicine, 462 Grider Street, Buffalo, NY 14215, United States

**Keywords:** atraumatic ankle fracture, Weber C fracture, bilateral fibular fracture, sequential fractures, comorbidities

## Abstract

We present the case of a 61-year-old female with bilateral sequential atraumatic Weber C ankle fractures occurring 2 years apart. Her medical history was notable for idiopathic neuropathy, balance disorder, morbid obesity, and vitamin D deficiency. She initially sustained a left-sided Weber C fracture requiring surgical fixation, followed by a similar atraumatic fracture on the contralateral side. Magnetic resonance imaging of the right ankle revealed a supra-syndesmotic fibular fracture with syndesmotic injury, prompting surgical intervention involving fibular osteotomy, locking plate fixation, and tightrope syndesmotic stabilization. To our knowledge, this is the first report of sequential, atraumatic Weber C ankle fractures in the literature. This case highlights the importance of recognizing non-traumatic etiologies of high fibular fractures in patients with impaired balance, altered biomechanics, and metabolic bone disease.

## Introduction

Ankle fractures are among the most common fractures worldwide, affecting individuals across all age groups and genders [1–3]. Though the incidence of ankle fractures peaks in adolescence, the occurrence of these injuries increases with age, especially among women [4]. Atraumatic ankle fractures are gaining recognition due to their association with underlying medical conditions. These injuries occur without trauma and are attributed to factors such as poor bone quality, neurological disorders, endocrine imbalances, and chronic inflammation [4, 5]. Atraumatic fractures of the ankle may be influenced by a combination of degenerative joint disease, decreased bone mineral density, and soft tissue dysfunction which may increase susceptibility to stress fractures and tendon injuries [6, 7].

The Weber classification system for lateral malleolus fractures is widely utilized to categorize ankle fractures based on the level of injury to the fibula and the integrity of the syndesmosis. Supra-syndesmotic fractures, or “Weber C” injuries are associated with high-energy trauma in young adults and are even more rarely associated with elderly women and low-energy trauma or the lack of traumatic insult [5]. Herein, we discuss the surgical treatment and post-operative outcome of a case of bilateral atraumatic Weber C ankle fractures sustained 2 years apart. The case of a 61-year-old female with multiple comorbidities who developed an atraumatic right Weber C fracture 2 years after sustaining an atraumatic left Weber C fracture.

## Case report

**Figure 1:**
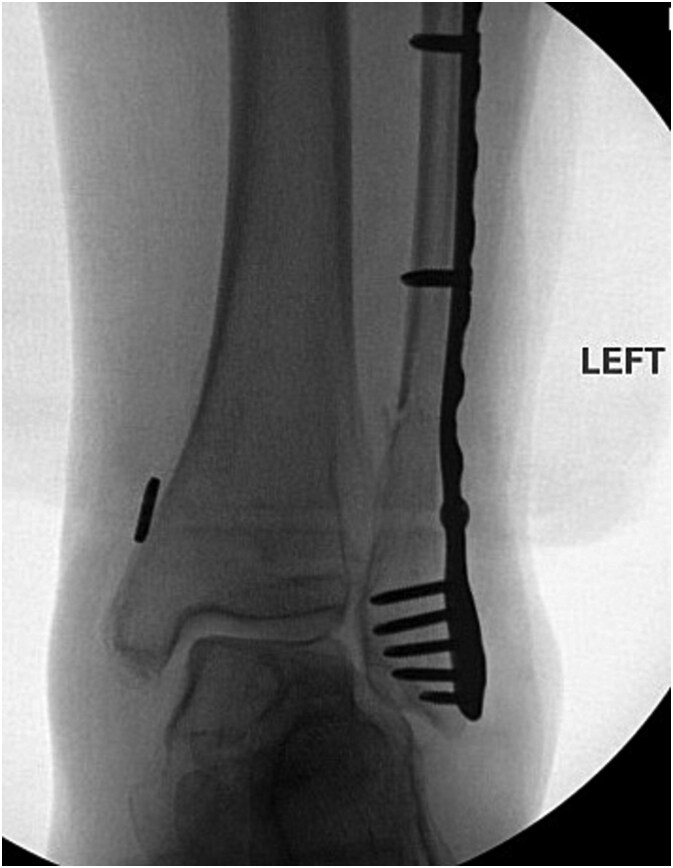
Anterior–posterior post-operative X-ray demonstrating fixation of left Weber C fibular fracture.

**Figure 2:**
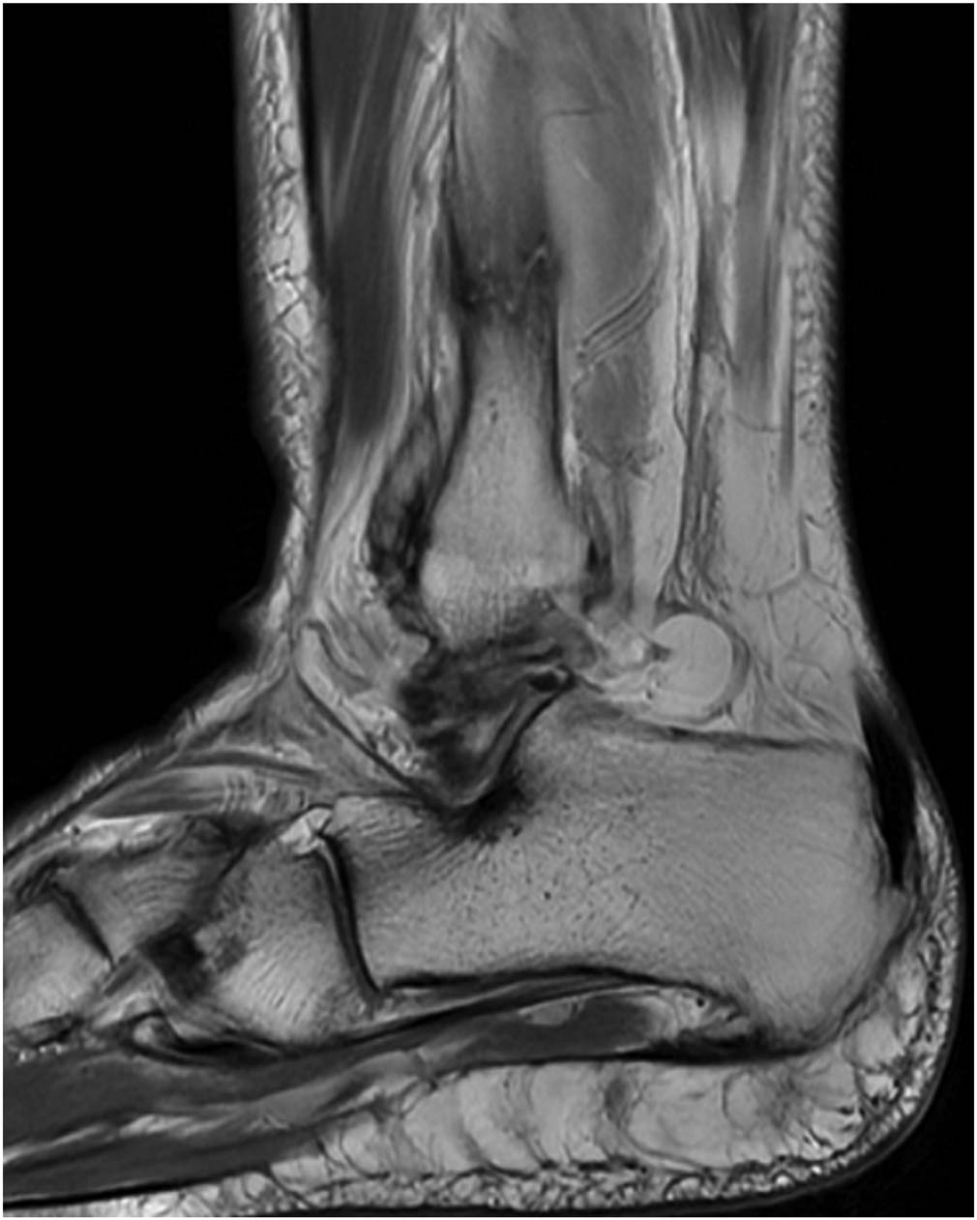
MRI demonstrating acute Weber C fibular fracture with partial-thickness tearing of the syndesmotic membrane of the right lower extremity.

The patient is a 61-year-old female with a past medical history of idiopathic neuropathy with balance disorder, morbid obesity, and vitamin D deficiency presented to the clinic after she sustained an atraumatic injury to the right ankle. Notably, the patient required surgical treatment 2 years prior for an atraumatic left Weber C lateral malleolus fracture with Stage 3 posterior tibialis tendonitis and pes planus deformity (Fig. 1). Radiographs obtained at the time of injury to her right ankle showed no acute osseous abnormalities or dislocations and she was given a tall controlled ankle motion (CAM) boot. Magnetic resonance imaging (MRI) was later obtained demonstrating an acute Weber C fibular fracture with partial-thickness tearing of the syndesmotic membrane (Fig. 2). She was diagnosed with a right ankle lateral malleolus Weber C stress fracture. Patient initially elected for nonsurgical treatment but after a week with stable pain with limited function, she elected for surgical intervention a week after injury.

With the patient in supine position, a longitudinal incision was made across the right lateral malleolus. A lateral malleolar Weber C ankle fracture with transverse comminution was identified. Subsequently, a fibular osteotomy was performed with a mallet and osteotome. Incarcerated nonviable bone, periosteum, and soft tissue debride was extracted.

**Figure 3:**
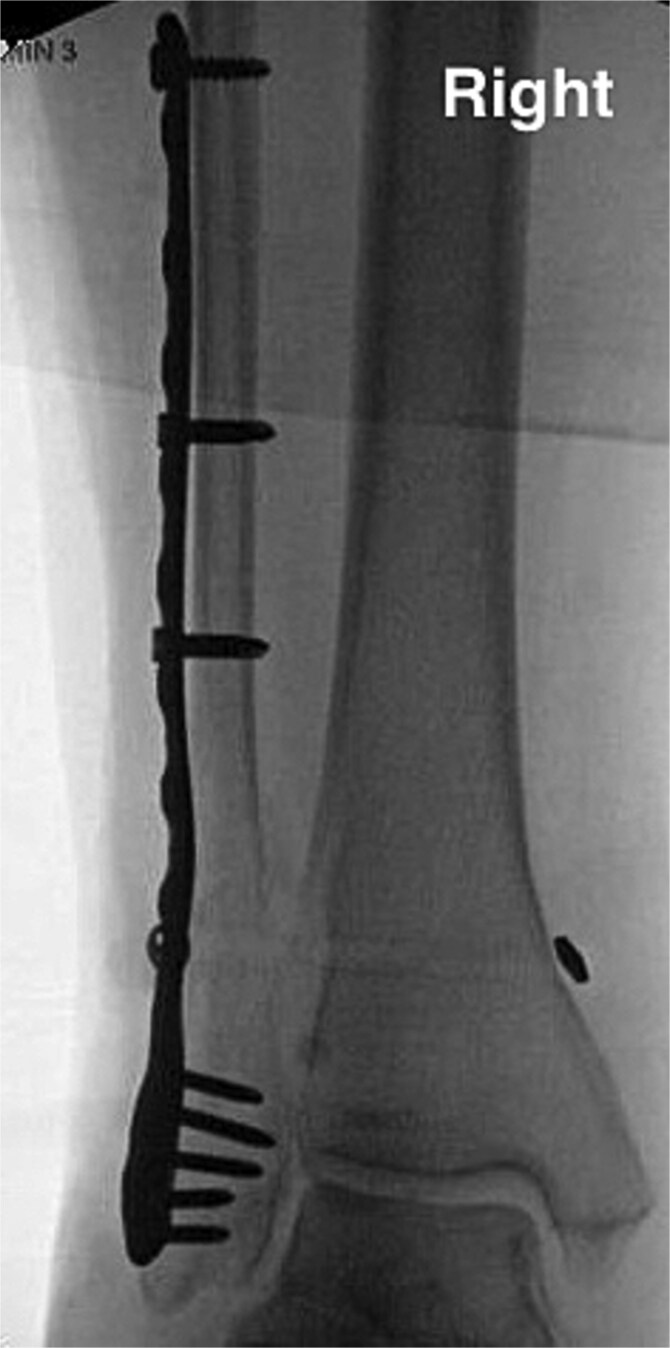
Anterior–posterior post-operative X-ray demonstrating fixation of right Weber C fibular fracture.

The lateral malleolus Weber C fracture was subsequently reduced to anatomical position. A locking stainless-steel plate was pre-contoured for the patient’s fibula and placed along the right lateral malleolus. Two 3.5 mm fully threaded bi-cortical locking screws were placed above the fracture and another 3.5 mm fully threaded bi-cortical locking screw was placed at the proximal end of the plate. A total of five 2.7-mm uni-cortical locking screws were used for fixation below the fracture site. Fluoroscopic images of the right ankle were taken in three views with simulated weight bearing in order to confirm anatomic alignment of the lateral malleolus Weber C fracture with proper plate and screw fixation. The syndesmosis was stressed in order to assess deltoid ligament function which showed widening. The distal tibiofibular joint was debrided and a king tong clamp was placed across the tibia and fibula through the fibular plate. Dorsiflexion of the ankle and hanging the posterior heel off a bump was done to avoid any anterior drawer effects on the ankle; following such, a tightrope was placed. Following final imaging (Fig. 3), the wound was then closed in layers.

An Association of Osteosynthesis (AO) splint using 5-in. plaster was applied with the ankle in neutral dorsiflexion. Patient was advised not to bear weight on her left leg for 10 weeks after the operation due to her polyneuropathy. After 2 weeks post-operation, a short leg cast was applied. On Week 6, follow-up radiographs were taken and a new short leg cast was applied. On Week 10, the patient was transitioned to CAM boot with weight-bearing-as-tolerated precautions, compression stockings, and range of motion to the ankle. On Week 14, the patient was advised to progress to normal shoes as her pain, swelling, and function would allow.

## Discussion

While previous literature, such as the case reported by Murray *et al*. has documented bilateral atraumatic fibular fractures classified as Weber C, no prior reports have detailed the occurrence of sequential fractures in the same patient [8]. This distinction is notable as it underscores the heightened risk for these fractures to develop sequentially in patients with compromised bone health and altered biomechanics.

Atraumatic fractures, particularly in the ankle, are an emerging concern in orthopedic practice. The literature on atraumatic fractures in older adults has primarily focused on osteoporosis and metabolic bone diseases as contributing factors [9], though other factors such as neuropathy and balance disorders may also play a significant role [10]. Whether in isolation or occurring concomitantly, these factors may predispose to fracture in the absence of direct trauma [11]. Notably, Knapp and Constant reported tendon dysfunction to be a contributing etiology as altered joint mechanics yield atypical stresses onto the ankle joint [12]. Further research is needed to elucidate the complex interplay of these factors and develop targeted interventions to prevent atraumatic fractures in high-risk populations.

## Conclusion

This case highlights a rare presentation of sequential, atraumatic Weber C ankle fractures in an elderly female with multiple comorbidities. The unique progression of bilateral high fibular stress fractures occurring 2 years apart underscores the importance of recognizing non-traumatic mechanisms of injury in patients with altered biomechanics and compromised bone health. Surgical management with fibular osteotomy, locking plate fixation, and syndesmotic stabilization provided successful outcomes in restoring alignment and function. Clinicians should maintain a high index of suspicion for atraumatic ankle fractures in patients with idiopathic neuropathy or balance impairment, even in the absence of significant trauma.
